# Supplementary material for: Cortex-wide transcranial localization microscopy with fluorescently labeled red blood cells
Source: Nat Commun. 2024 Apr 25;15:3526. doi: 10.1038/s41467-024-47892-3 (PMC11045747; doi:10.1038/s41467-024-47892-3)
Supplement: Supplementary file 1 — Supplementary Information [file 41467_2024_47892_MOESM1_ESM.pdf]

Supplementary Information for

Cortex-wide transcranial localization microscopy with fluorescently labeled red blood cells

Quanyu Zhou,[1,2,†] Chaim Glück,[1,3,†] Lin Tang,[1,2] Lukas Glandorf,[1,2] Jeanne Droux,[3,4] Mohamad El Amki,[3,4] Susanne Wegener,[3,4] Bruno Weber,[1,3] Daniel Razansky,[1,2,3,*] and Zhenyue Chen,[1,2,*]

[1]Institute of Pharmacology and Toxicology, Faculty of Medicine, University of Zurich, Zurich, Switzerland

[2]Institute for Biomedical Engineering, Department of Information Technology and Electrical Engineering, ETH Zurich, Zurich, Switzerland

[3]Zurich Neuroscience Center, Zurich, Switzerland

[4]Department of Neurology, University Hospital and University of Zurich, Zurich, Switzerland

† These authors contributed equally

* Correspondence: daniel.razansky@uzh.ch; zhenyue.chen@uzh.ch

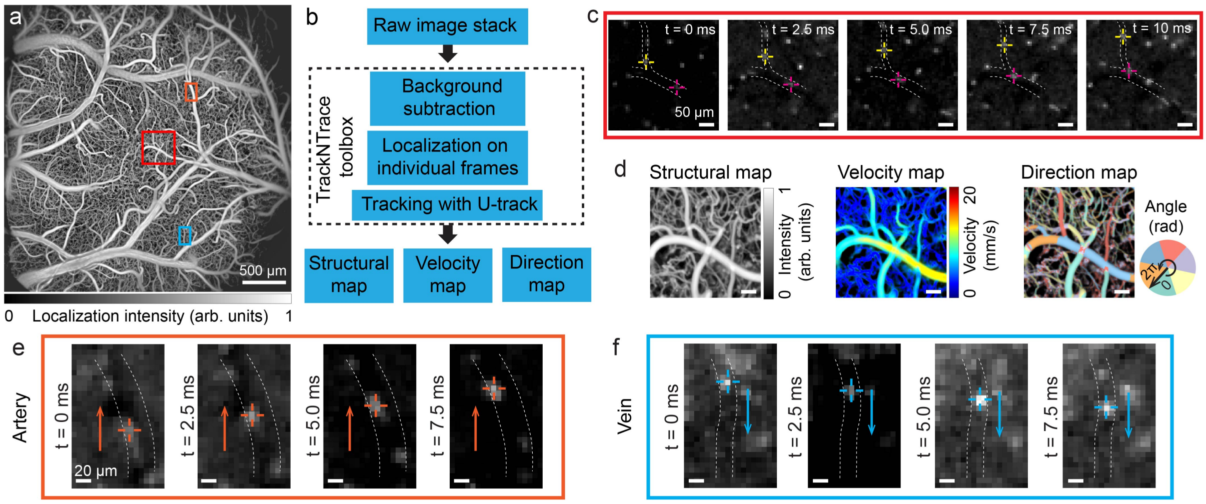

Supplementary Figure 1. Principle of localization-based image reconstruction and differentiation of arteries and veins based on flow direction. (a) Localization reconstructed structural map of the mouse brain. **(b)** Image processing pipeline of localization-based reconstruction. **(c)** Time-lapse images of stained RBCs from a ROI outlined by the red square in **a**. **(d)** Reconstructed structural, velocity, direction maps of the same ROI. **(e, f)** Time-lapse images of a representative artery and vein marked with rectangles in **a**, whereas opposite flow directions (labeled with arrows) were observed.

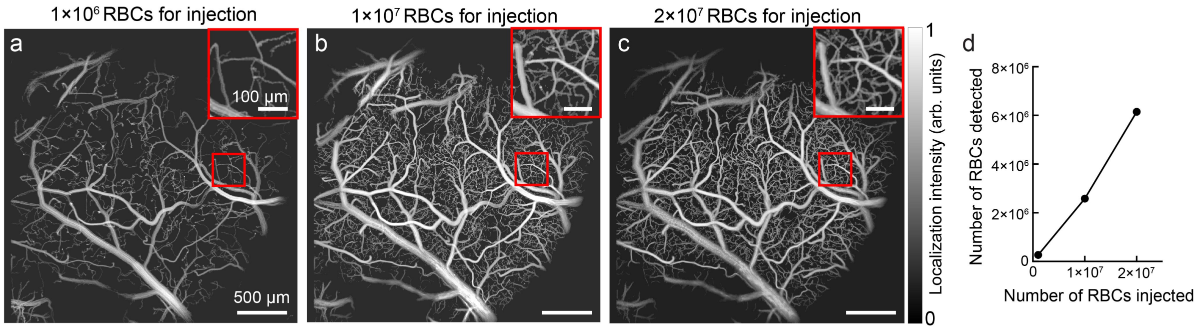

Supplementary Figure 2. Impact of the number of injected stained RBCs on image quality of RBC-aided WFLM. (a-c) Localization reconstructed structural maps of cerebral vasculature in a C57BL/6 mouse implanted with cranial window post intravenous injection of 1×10^6, 1×10^7, 2×10^7 stained RBCs, respectively. The widefield recording lasted for 30 s. Zoom-in views of a selected ROI were shown on the top. **(d)** Relationship between the number of detected RBCs across the FOV and the number of injected stained RBCs. Source data of Supplementary Fig. 2d are provided as a Source Data file.

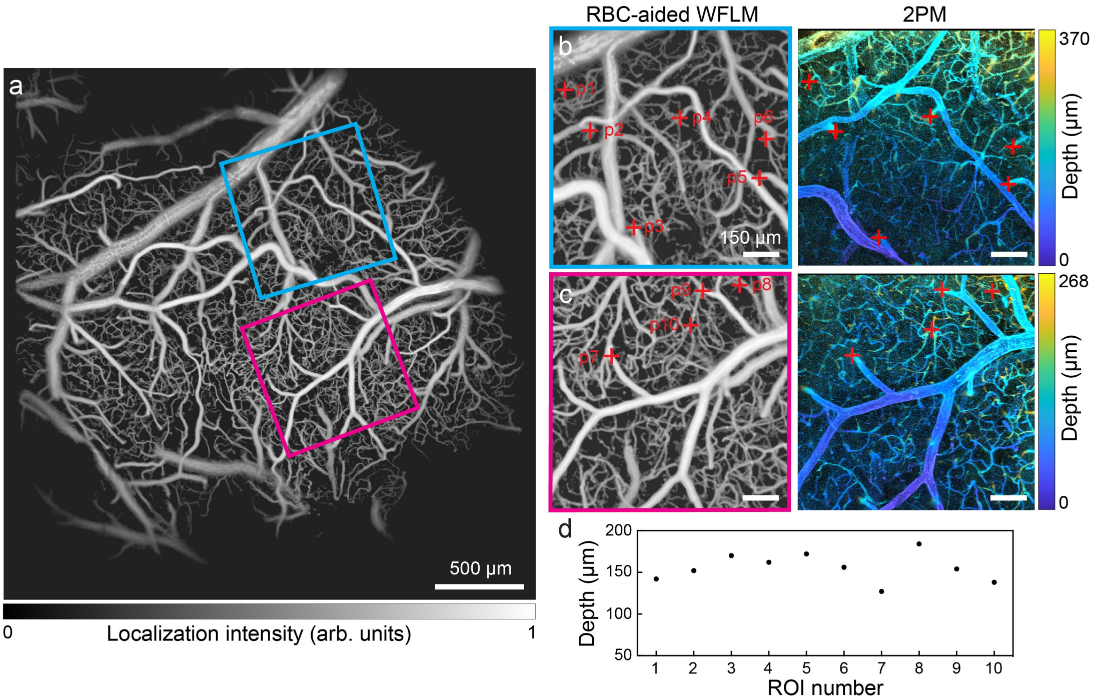

Supplementary Figure 3. Imaging depth validation of RBC-aided WFLM with two-photon laser scanning microscopy (2PM). (a) Localization reconstructed structural map of the mouse brain. **(b, c)** Comparison of the same ROIs (marked with squares in **a**) captured with RBC-aided WFLM and 2PM, respectively. The 2PM imaging was conducted on the same mouse brain *ex vivo*, providing a color-encoded depth map rendered from axial scanning at a step size of 2 µm. **(d)** Measured depth relative to the brain surface for 10 selected vessels (marked with crosses in **b, c**) that were detected with both methods. Source data of Supplementary Fig. 3d are provided as a Source Data file.

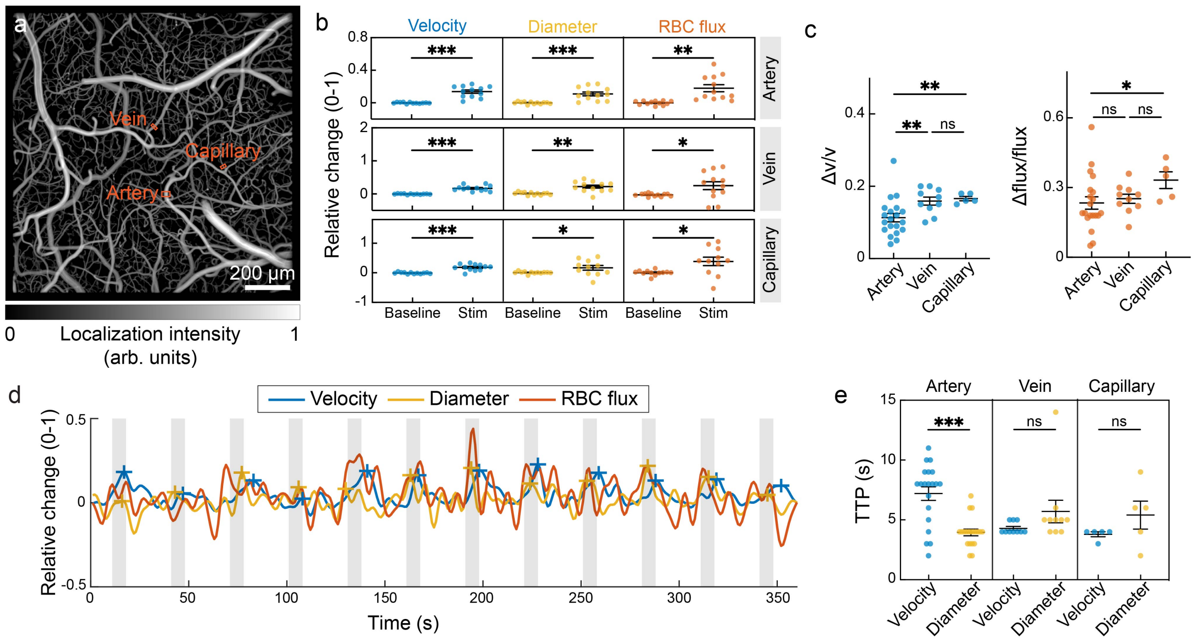

Supplementary Figure 4. Multiparametric hemodynamic measurements in various vessel types during hindpaw stimulation. **(a)** Localization structural map of brain microcirculation in a representative C57BL/6 mouse. **(b)** Relative change of hemodynamic parameters (velocity, diameter, and RBC flux) under the baseline and peak response time for the selected artery, vein, and capillary (labeled with rectangles in **a**) across n = 12 stimulation cycles. The hemodynamic parameters in baseline were averaged over a 10 s baseline period. **(c)** Fractional changes in velocity and RBC flux among vessel types (n = 20 arteries, n = 10 veins, and n = 5 capillaries). **(d)** Temporal profiles of velocity, diameter, and RBC flux in the selected artery (labeled in **a**) over 12 stimulation cycles. **(e)** Time-to-peak (TTP) values for velocity and diameter changes across vessel types (n = 20 arteries, n = 10 veins, and n = 5 capillaries). Two-tailed paired t-test and Wilcoxon test (if data didn't pass normality test) was applied in **b** and **e** while two-tailed Mann-Whitney test was used in **c** (ns, not significant, $*P < 0.05$, $**P<0.01$, $***P < 0.001$). Source data of Supplementary Figs. 4b-e are provided as a Source Data file.

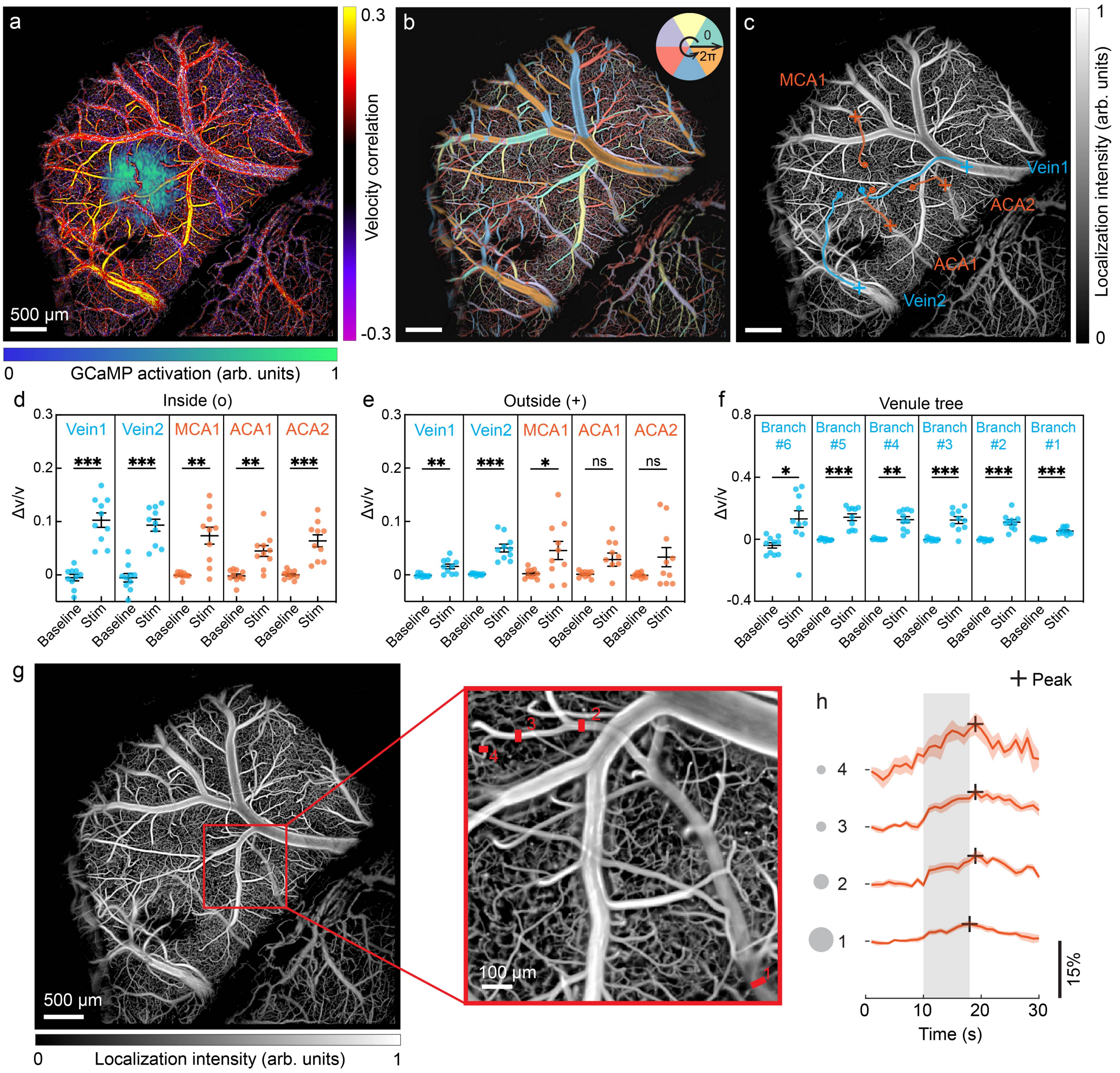

Supplementary Figure 5. Neuro-vascular activation patterns and statistical analysis of velocity change in selected vessel segments in mice under hindpaw stimulation. (a) Overlap of the GCaMP activation map and velocity correlation map for a Thy1-GCaMP6f mouse under right hindpaw stimulation. **(b)** Color-encoded flow direction map where arteries and veins are distinguishable. From the map, five vessel segment pairs were chosen both inside and outside the GCaMP activation region in **c**. **(d, e)** Fractional change of flow velocity under baseline and peak response time for selected veins and arteries inside and outside GCaMP activation region (marked in **c**) across n = 10 stimulation cycles. **(f)** Fractional change of flow velocity under baseline and peak response time for the selected venule tree (labeled in Fig. 3f) across n = 10 stimulation cycles. **(g)** Vessel segments selected along an arterial tree with averaged velocity time courses depicted in **h**. Data are presented as the mean ± s.e.m. Two-tailed paired t-test and Wilcoxon test (ns, not significant, *$P < 0.05$, **$P<0.01$, ***$P < 0.001$). Source data of Supplementary Figs. 5d-f, h are provided as a Source Data file.

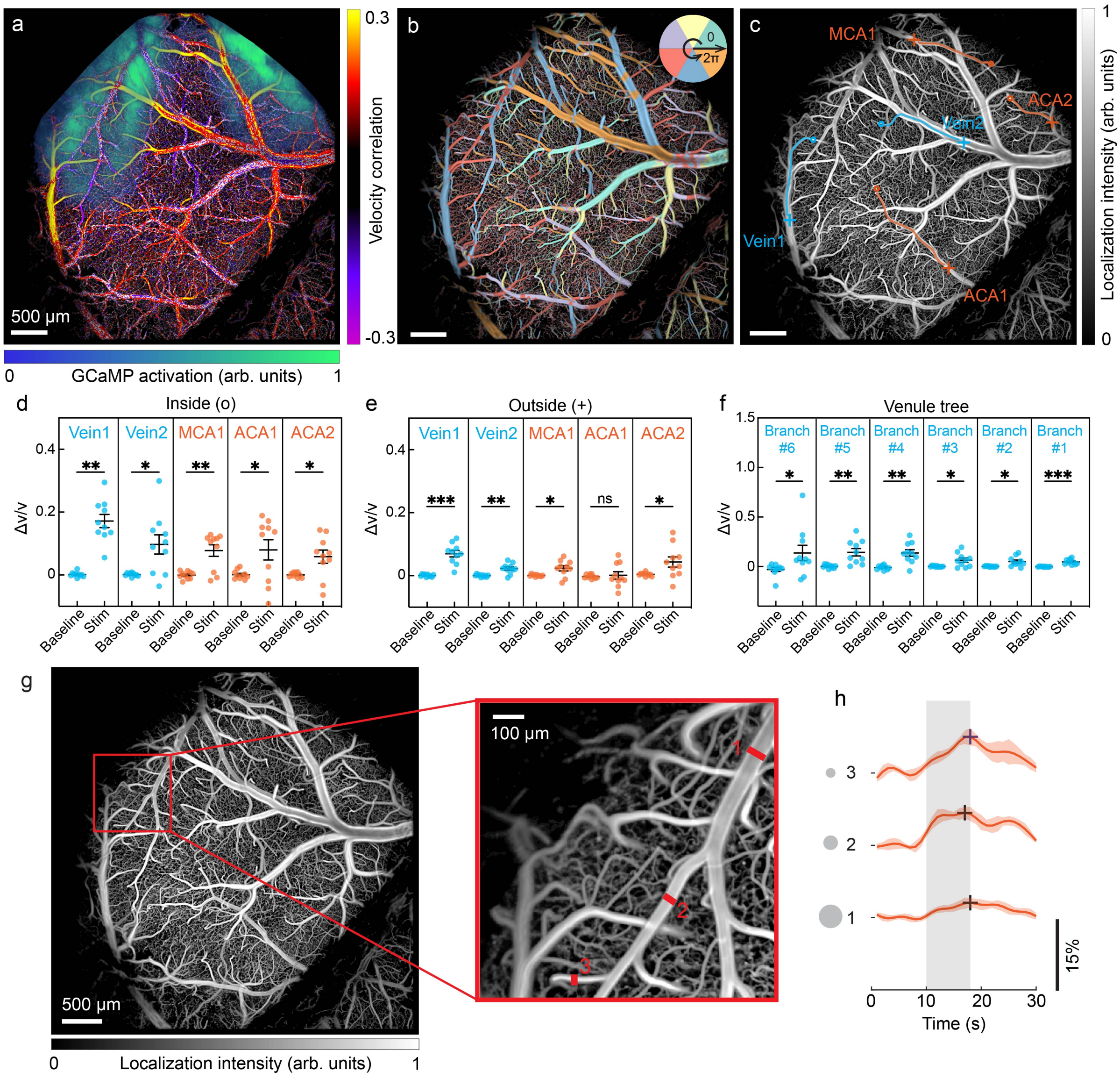

Supplementary Figure 6. Neuro-vascular activation patterns and statistical analysis of velocity change in selected vessel segments in mice under whisker stimulation. (a) Overlap of the GCaMP activation map and velocity correlation map for a Thy1-GCaMP6f mouse under right whisker stimulation. **(b, c)** Color-encoded flow direction map and selection of vessel segments inside and outside the GCaMP activation region. **(d, e)** Fractional change of flow velocity under baseline and peak response time for selected veins and arteries inside and outside GCaMP activation region (marked in **c**) across n = 10 stimulation cycles. **(f)** Fractional change of flow velocity under baseline and peak response time for the selected venule tree (labeled in Fig. 3m) across n = 10 stimulation cycles. **(g)** Vessel segments selected along an arterial tree with averaged velocity time courses depicted in **h**. Data are presented as the mean ± s.e.m. Two-tailed paired t-test and Wilcoxon test (ns, not significant, *$P < 0.05$, **$P<0.01$, ***$P < 0.001$). Source data of Supplementary Figs. 6d-f, h are provided as a Source Data file.

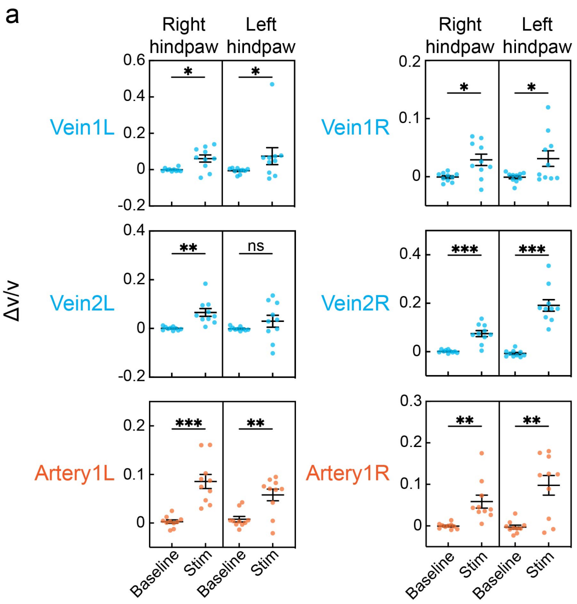

Supplementary Figure 7. Statistical analysis of velocity change in selected vessel segments measured transcranially in mice under bilateral hindpaw stimulation. (a) Fractional change of flow velocity under baseline and peak response time for selected veins and arteries situated at both hemispheres (marked in Fig. 4a) across $n = 10$ stimulation cycles. Two-tailed paired t-test and Wilcoxon test (ns, not significant, $*P < 0.05$, $**P<0.01$, $***P < 0.001$). Source data are provided as a Source Data file.

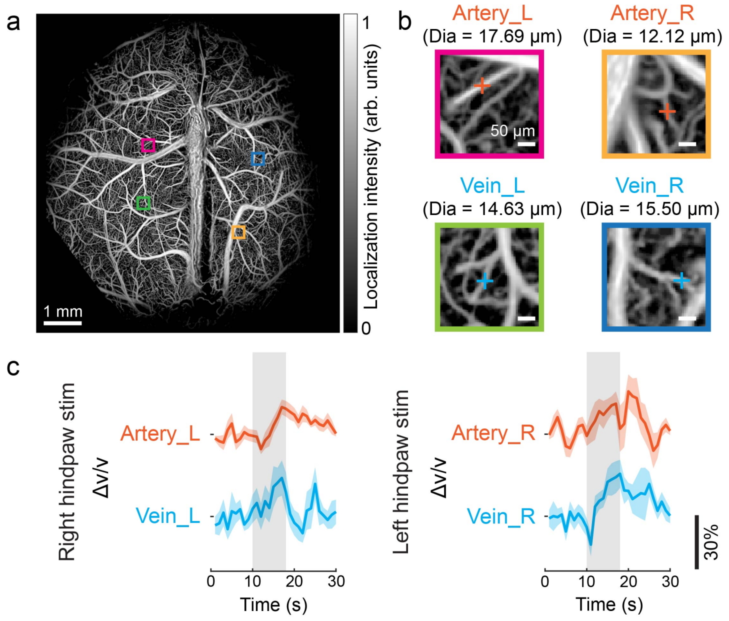

Supplementary Figure 8. Transcranial imaging of hindpaw stimulation-evoked hemodynamic changes in arterioles and venules in murine brain. (a) Localization structural map of brain microcirculation in a Thy1-GCaMP6f mouse. **(b)** Zoom-in views of four ROIs selected from both hemispheres, which are indicated with squares in **a**. Representative arterioles and venules were selected from ROIs with vessel diameters labeled. **(c)** Temporal profiles of flow velocity change within the selected arterioles and venules in response to bilateral hindpaw stimulation. Source data of Supplementary Fig. 8c are provided as a Source Data file.

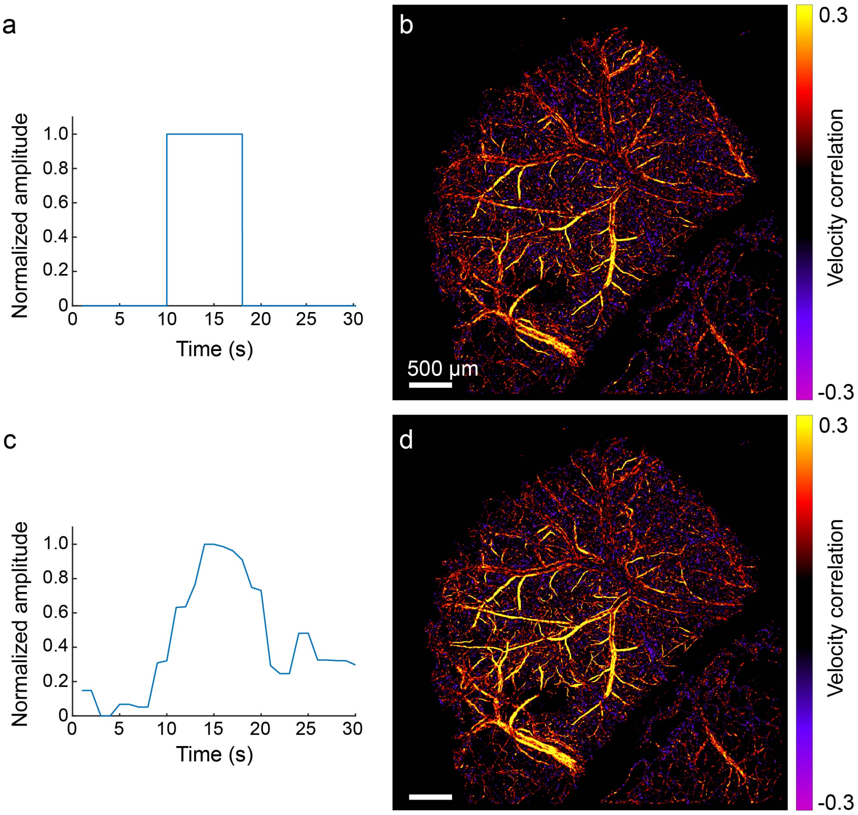

Supplementary Figure 9. Calculation of velocity correlation map. (a) Single-cycle stimulation pattern represented with a boxcar function. **(b)** Velocity correlation map generated by pixelwise computation of the Pearson's correlation coefficient between the stimulation pattern and velocity time course. **(c)** Local averaged hemodynamic response curve derived from averaging the velocity time courses of pixels with correlation coefficients exceeding the 90% quantile. **(d)** Enhanced velocity correlation map produced by replacing the stimulation pattern with local averaged hemodynamic response curve in the calculation. Source data of Supplementary Figs. 9a, c are provided as a Source Data file.

Supplementary Table 1. Imaging conditions for experiments.

	**RBC-aided WFLM**						**Epi-fluorescence calcium imaging**	
	Frame rate (Hz)	Magnification ratio	NA	FOV (mm^2)	Pixel size (μm)	FWHM of the smallest vessel structure (μm)	Frame rate (Hz)	Resolution (μm)
Fig. 1	400	1.50	0.04	7.39×7.39	7.33	4.90	No calcium recording performed	
Fig. 2	833	2.75	0.08	4.03×4.03	4.00	2.75	No calcium recording performed	
Fig. 3	400	1.50	0.04	7.39×7.39	7.33	4.90	40	18.60
Fig. 4	400	0.86	0.04	12.94×12.94	12.83	9.92	20	18.60

Abbreviations: NA, numerical aperture. FOV, field of view; FWHM, full-width-at-half-maximum.
