# Supplementary material for: Cortex-wide transcranial localization microscopy with fluorescently labeled red blood cells
Source: Nat Commun. 2024 Apr 25;15:3526. doi: 10.1038/s41467-024-47892-3 (PMC11045747; doi:10.1038/s41467-024-47892-3)
Supplement: Supplementary file 2 — Reporting Summary [file 41467_2024_47892_MOESM2_ESM.pdf]

nature portfolio

Corresponding author(s): Daniel Razansky; Zhenyue Chen

Last updated by author(s): Apr 1, 2024

Reporting Summary

Nature Portfolio wishes to improve the reproducibility of the work that we publish. This form provides structure for consistency and transparency in reporting. For further information on Nature Portfolio policies, see our Editorial Policies and the Editorial Policy Checklist.

Statistics

For all statistical analyses, confirm that the following items are present in the figure legend, table legend, main text, or Methods section.

n/a	Confirmed	
☐	☒	The exact sample size (*n*) for each experimental group/condition, given as a discrete number and unit of measurement
☐	☒	A statement on whether measurements were taken from distinct samples or whether the same sample was measured repeatedly
☐	☒	The statistical test(s) used AND whether they are one- or two-sided<br>*Only common tests should be described solely by name; describe more complex techniques in the Methods section.*
☒	☐	A description of all covariates tested
☐	☒	A description of any assumptions or corrections, such as tests of normality and adjustment for multiple comparisons
☐	☒	A full description of the statistical parameters including central tendency (e.g. means) or other basic estimates (e.g. regression coefficient) AND variation (e.g. standard deviation) or associated estimates of uncertainty (e.g. confidence intervals)
☐	☒	For null hypothesis testing, the test statistic (e.g. *F*, *t*, *r*) with confidence intervals, effect sizes, degrees of freedom and *P* value noted<br>*Give P values as exact values whenever suitable.*
☒	☐	For Bayesian analysis, information on the choice of priors and Markov chain Monte Carlo settings
☒	☐	For hierarchical and complex designs, identification of the appropriate level for tests and full reporting of outcomes
☐	☒	Estimates of effect sizes (e.g. Cohen's *d*, Pearson's *r*), indicating how they were calculated

Our web collection on statistics for biologists contains articles on many of the points above.

Software and code

Policy information about availability of computer code

Data collection	1. Camware (version 4.12, PCO AG, Germany) was used for RBC-aided WFLM data collection.<br>2. ScanImage (r3.8.1, Janelia Research Campus) was used for two-photon laser scanning microscopy data collection.
Data analysis	1. TrackNTrace (version 1.03, https://github.com/scstein/TrackNTrace, 2016) was used for localization and tracking.<br>2. Custom MATLAB (R2020b, MathWorks, USA) codes were used for image reconstruction, which are provided on the Zenodo repository website at: https://doi.org/10.5281/zenodo.10663497.<br>3. The "sinefit" function (version 3.2.1, Peter Seibold, https://www.mathworks.com/matlabcentral/fileexchange/66793-sine-fitting, 2023) was used for sine fitting and Fourier analysis.<br>4. The "corrcoef" function with MATLAB (R2020b, MathWorks, USA) was used to calculate the Pearson's correlation coefficients.<br>5. The "vessel diameter.ijm" plugin (version 1.0) in ImageJ (version 1.54d, National Institutes of Health, USA) was used to quantify the vessel diameter.

For manuscripts utilizing custom algorithms or software that are central to the research but not yet described in published literature, software must be made available to editors and reviewers. We strongly encourage code deposition in a community repository (e.g. GitHub). See the Nature Portfolio guidelines for submitting code & software for further information.

Data

Policy information about availability of data

All manuscripts must include a data availability statement. This statement should provide the following information, where applicable:

- Accession codes, unique identifiers, or web links for publicly available datasets
- A description of any restrictions on data availability
- For clinical datasets or third party data, please ensure that the statement adheres to our policy

The main data supporting the finding of this study are available within the main text or Supplementary Information. Source data are provided with this paper. The raw datasets before image reconstruction are too large to be publicly shared, yet they are available for research purposes from the corresponding author upon request. Requests will be fulfilled within 4 weeks.

Research involving human participants, their data, or biological material

Policy information about studies with human participants or human data. See also policy information about sex, gender (identity/presentation), and sexual orientation and race, ethnicity and racism.

Reporting on sex and gender	Sex and gender were not considered in the study design since the main purpose of this study is to present a versatile toolset for functional neuroimaging.
Reporting on race, ethnicity, or other socially relevant groupings	This study did not require the consideration of socially constructed or socially relevant categories as variables.
Population characteristics	Not applicable
Recruitment	Not applicable
Ethics oversight	Not applicable

Note that full information on the approval of the study protocol must also be provided in the manuscript.

Field-specific reporting

Please select the one below that is the best fit for your research. If you are not sure, read the appropriate sections before making your selection.

☒ Life sciences ☐ Behavioural & social sciences ☐ Ecological, evolutionary & environmental sciences

For a reference copy of the document with all sections, see nature.com/documents/nr-reporting-summary-flat.pdf

Life sciences study design

All studies must disclose on these points even when the disclosure is negative.

Sample size	No sample-size calculation was performed since the study focused on the proof of concept of the proposed method. N = 1 mouse was used in Figure 1 and 2 to characterize the imaging performance of the method, which is expected to be similar on different animals. N = 5 mice were used for both hindpaw and whisker stimulations whereas representative results from one mouse were shown in Figure 3. N = 4 mice were used for hinpdaw stimulations whereas representative results from one mouse were shown in Figure 4. N = 1 mouse was used in Supplementary Figure 2 and Supplementary Figure 3 to assess the impact of stained red blood cell number on image quality and validate the imaging depth. For statistical analysis, different region of interests were chosen independently in the same mouse for data analysis with sample size labeled in the figure legend or main text.
Data exclusions	No data exclusion.
Replication	The system characterization experiments in Figure 1 and 2 were performed with N = 1 mouse for each. The stimulation experiments in Figure 3 and 4 were repeated with N = 5 and N= 4 mice respectively, which showed consistent results. Image quality evaluation and imaging depth validation experiment in Supplementary Figure 2 and Supplementary Figure 3 was performed with N = 1 mouse.
Randomization	Randomization was not relevant since no group comparison was performed in this proof of concept study.
Blinding	Blinding was not relevant since no group comparison was performed in this proof of concept study.

Reporting for specific materials, systems and methods

We require information from authors about some types of materials, experimental systems and methods used in many studies. Here, indicate whether each material, system or method listed is relevant to your study. If you are not sure if a list item applies to your research, read the appropriate section before selecting a response.

Materials & experimental systems

n/a	Involved in the study	
☒	☐	Antibodies
☒	☐	Eukaryotic cell lines
☒	☐	Palaeontology and archaeology
☐	☒	Animals and other organisms
☒	☐	Clinical data
☒	☐	Dual use research of concern
☒	☐	Plants

Methods

n/a	Involved in the study	
☒	☐	ChIP-seq
☒	☐	Flow cytometry
☒	☐	MRI-based neuroimaging

Animals and other research organisms

Policy information about studies involving animals; ARRIVE guidelines recommended for reporting animal research, and Sex and Gender in Research

Laboratory animals	In total, two C57BL/6 mice (19 weeks old and 38 weeks old, Envigo BMS B.V., Netherlands) and ten GCaMP6f mice (6-21 weeks old, C57BL/6J-Tg (Thy1-GCaMP6f) GP5.17Dkim/J, the Jackson Laboratory, USA) were used in the study.
Wild animals	No wild animals were used in the study.
Reporting on sex	Both male and female mice were used in the study including three female and nine male mice.
Field-collected samples	No field-collected samples were used in the study.
Ethics oversight	All animal experiments were performed in accordance with the Swiss Federal Act on Animal Protection and approved by the Cantonal Veterinary Office Zurich.

Note that full information on the approval of the study protocol must also be provided in the manuscript.

Plants

Seed stocks	Not applicable
Novel plant genotypes	Not applicable
Authentication	Not applicable
