# Supplementary material for: Cortex-wide transcranial localization microscopy with fluorescently labeled red blood cells
Source: Nat Commun. 2024 Apr 25;15:3526. doi: 10.1038/s41467-024-47892-3 (PMC11045747; doi:10.1038/s41467-024-47892-3)
Supplement: Supplementary file 4 — Description of Additional Supplementary Files [file 41467_2024_47892_MOESM4_ESM.pdf]

Title: Supplementary Movie 1
Description: Hemodynamic responses in the murine brain under hindpaw stimulation across various vessel types captured with RBC-aided WFLM.

Title: Supplementary Movie 2
Description: Simultaneous neuro-vascular activation mapping of the murine brain under whisker stimulation captured with RBC-aided WFLM.
